# Chinese herbal medicine therapy and the risk of mortality for chronic hepatitis B patients with concurrent liver cirrhosis: a nationwide population-based cohort study

**DOI:** 10.18632/oncotarget.24383

**Published:** 2018-04-06

**Authors:** Tzung-Yi Tsai, Tsung-Hsing Hung, Hanoch Livneh, I-Hsin Lin, Ming-Chi Lu, Chia-Chou Yeh

**Affiliations:** ^1^ Department of Medical Research, Dalin Tzuchi Hospital, The Buddhist Tzuchi Medical Foundation, Chiayi 62247, Taiwan; ^2^ Department of Environmental and Occupational Health, College of Medicine, National Cheng Kung University, Tainan 70428, Taiwan; ^3^ Department of Nursing, Tzu Chi College of Technology, Hualien 97004, Taiwan; ^4^ Division of Gastroenterology, Dalin Tzu Chi Hospital, Buddhist Tzu Chi Foundation, Chiayi 62247, Taiwan; ^5^ School of Medicine, Tzu Chi University, Hualien 97004, Taiwan; ^6^ Rehabilitation Counseling Program, Portland State University, Portland, OR 97207-0751, USA; ^7^ School of Post-Baccalaureate Chinese Medicine, Tzu Chi University, Hualien 97004, Taiwan; ^8^ Division of Allergy, Immunology and Rheumatology, Dalin Tzuchi Hospital, The Buddhist Tzuchi Medical Foundation, Chiayi 62247, Taiwan; ^9^ Department of Chinese Medicine, Dalin Tzuchi Hospital, The Buddhist Tzuchi Medical Foundation, Chiayi 62247, Taiwan

**Keywords:** chronic hepatitis B, liver cirrhosis, mortality, Chinese herbal medicine, cohort study

## Abstract

Chronic hepatitis B (CHB) is increasingly recognized as a public health problem in Taiwan. After affected patients are diagnosed with contaminant liver cirrhosis (LC), adverse clinical outcomes, especially death, are common. This study aimed to investigate the effect of Chinese herbal medicine (CHM), an essential branch of Traditional Chinese medicine (TCM), on the mortality risk among CHB patients with contaminant LC. This longitudinal cohort study used the Taiwanese National Health Insurance Research Database to identify 1522 patients 20–70 years of age with newly diagnosed CHB with LC during 1998–2007. Among them, 508 (33.37%) had received CHM products after the onset of CHB (CHM users), and the remaining 1014 patients (66.63%) were designated as a control group (non-CHM users). All enrollees were followed until the end of 2012 to determine deaths during the study period. We applied the Cox proportional hazards regression model to compute the hazard ratio for the association of CHM use and the subsequent risk of death. During the follow-up period, 156 CHM users and 493 non-CHM users died. After controlling for potential confounders, CHM users were found to have a significantly reduced risk of death compared with non-CHM users by 56%, and the effect was predominantly observed among those treated with CHM for > 180 days. CHM therapy lowered the risk of death among CHB patients with contaminant LC, which supported CHM might provide further treatment options for those with chronic liver diseases.

## INTRODUCTION

Ascites, hepatic encephalopathy, and esophageal variceal bleeding are the 3 major complications of cirrhosis. The presence of these complications is a sign of decompensated liver cirrhosis and usually indicates a poor prognosis. In Taiwan, the most common etiology of liver cirrhosis is chronic hepatitis B (CHB) [1, 2], and most hepatitis B virus (HBV) infections occur following vertical transmission. In recent decades, despite the improvement of antiviral therapy, the burden of viral hepatitis has remained considerable. For example, Stanaway and colleagues indicated that the number of deaths worldwide attributable to viral hepatitis increased from 0.89 million deaths, in 1990, to 1.45 million deaths in 2013, an increase of 63% [3], suggesting that HBV places a tremendous burden on patients, their families, and the healthcare system.

Chinese herbal medicine (CHM) is the most commonly used alternative medicine for patients with chronic diseases, and several investigators suggested that this approach could improve clinical outcomes or quality of life. A recent 15-year cohort study of 112,458 patients with vertigo showed that those receiving CHM had a significantly lower risk (36%) of stroke than did those who did not receive CHM [4]. Another randomized controlled trial conducted among 352 individuals with chronic obstructive pulmonary disease compared the effectiveness of combining conventional Western medicine and several Chinese herbal products for 1 year [5], and reported that integrating CHM and Western medicine significantly improved pulmonary function, quality of life, and psychological health (mood and depression). Additionally, in a prior study, we found that the use of CHM was associated with a significantly reduced risk of hepatocellular carcinoma (HCC) in patients with CHB [6]. These findings indicated that the integration of CHM into allopathic clinical practice is likely to contribute to favorable outcomes.

We conducted a comprehensive literature review and discovered that no studies have been reported that identified the effect of CHM on the risk of mortality for CHB patients with concurrent liver cirrhosis (LC). Given that the liver disease is widespread in Taiwan, to prevent the corresponding damages, the investigation of the long-term effect of alternative treatments, specifically CHM, for the affected patients is important. More specifically, this study aimed to identify the effect of CHM on the risk of morality among CHB patients using a nationwide population-based database.

## RESULTS

We identified 1522 patients with CHB and contaminant LC between 1998 and 2007. Of these, 508 received CHM and 1014 were non-CHM users. Table 1 shows the basic characteristics of the 2 groups. Compared with non-CHM users, those who received CHM services were more likely to be female and younger (< 50 years), and to have a lower score *of Charlson* Comorbidity Index (CCI) (all *P* < 0.01) (Table 1).

**Table 1:** Demographic data and comorbidity comparisons

Variables	CHM nonusers	CHM users	*P*
	(*n* = 1014)	(*n* = 508)	
Age, (years)			0.003
≤ 50	461 (45.5)	255 (50.2)	
➢ 50	553 (54.5)	253 (49.8)	
Mean (Standard Deviation)	51.3 (11.1)	49.6 (11.2)	
Sex			0.001
Female	251 (24.8)	166 (32.7)	
Male	763 (75.2)	342 (67.3)	
Monthly income			0.27
Low	382 (37.7)	182 (35.8)	
Median	563 (55.5)	280 (55.1)	
High	69 (6.8)	46 (9.1)	
Residential area			0.93
Urban	560 (55.2)	277 (54.5)	
Suburban	166 (16.4)	82 (16.1)	
Rural	288 (28.4)	149 (29.3)	
CCI			< 0.001
Mean (SD)	19.73 (19.2)	15.30 (16.2)	

**Figure 1:**
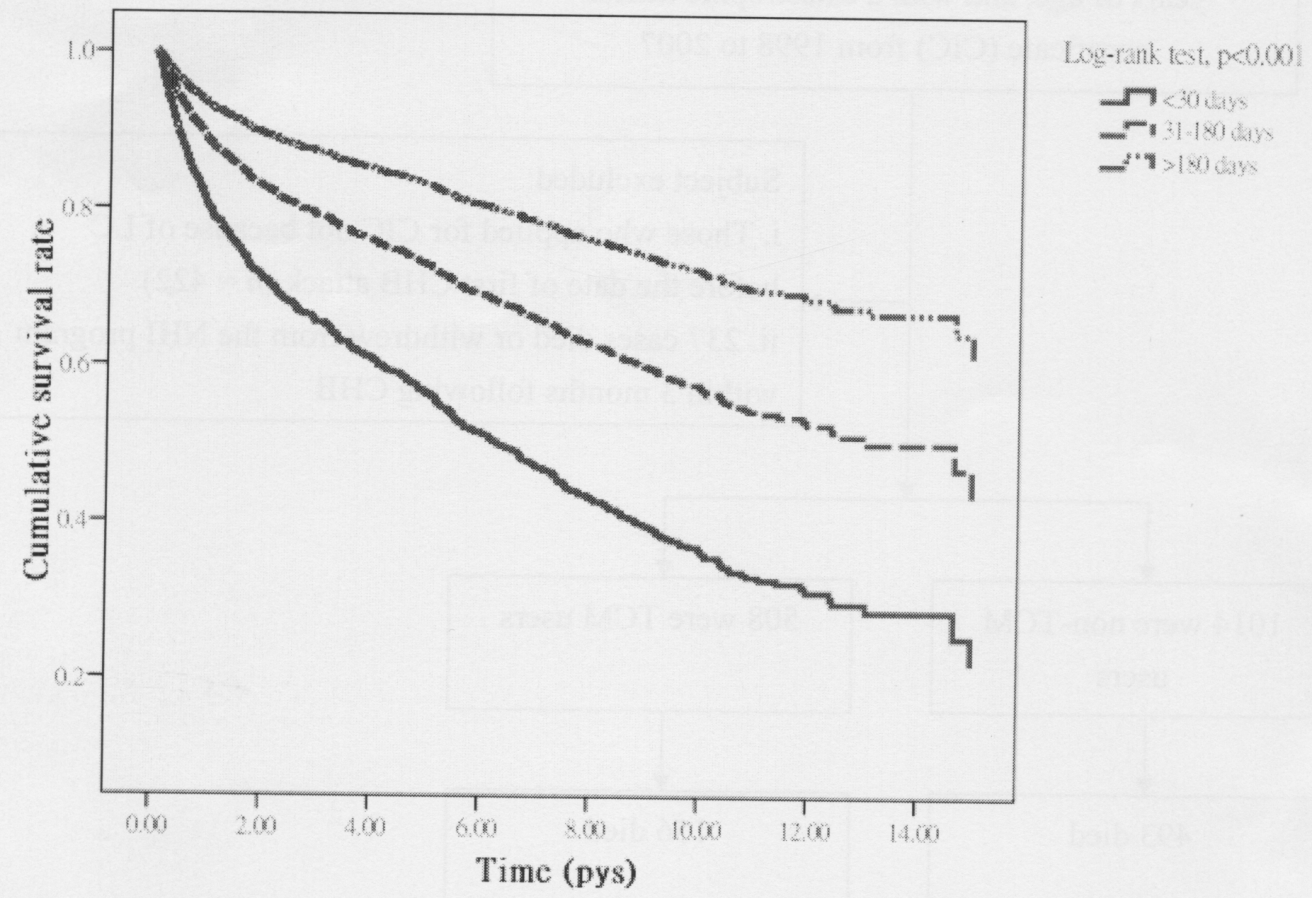
Survival rate according to TCM use status, all-cause mortality *P* is based on the log-rank test across 3 groups.

Among all eligible subjects, 649 deaths occurred, including 493 non-CHM users and 156 CHM users, during the follow-up periods of 3997.98 and 3644.58 person–years (PY), respectively. The mortality rate was lower in CHM users than in non-CHM users (42.80 vs 123.31, respectively, per 1000 PY), with an adjusted hazard ratio [3] of 0.44 (95% confidence interval [CI]: 0.36–0.52) (Table 2). Of note, patients who used CHM treatments for > 180 days had a 67% decreased risk of death (95% CI: 0.25–0.42). Based on the Kaplan–Meier survival curve and log-rank test results, a statistically significant difference regarding the survival rate was observed across the 3 groups during the 15-year follow-up period. Those who received CHM treatments for > 180 days had a significantly lower mortality rate than those who did not receive CHM (*P* < 0.001) (Figure 1).

**Table 2:** Crude and adjusted HR of HCC for CHB patients with and without CHM usage

Patient group	Event	PY	Incidence	Crude HR(95% CI)	Adjusted HR^*^(95% CI)
CHM nonusers	493	3997.98	123.31	1	1
CHM users	156	3644.58	42.80	0.35 (0.33–0.46)	0.44 (0.36–0.52)
CHM use within 30–180 days	94	1623.47	57.90	0.47 (0.41–0.64)	0.56 (0.45–0.70)
CHM use lasted for > 180 days	62	2021.11	30.68	0.25 (0.21–0.35)	0.33 (0.25–0.42)

Incidence rate is per 1000 PY.

^*^Model adjusted for age, sex, urbanization level, monthly income, and CCI scores.

Table 3 presents results from an analysis that stratified patients by age and sex. We found that females who received CHM had a lower mortality rate than did their non-CHM counterparts (27.10 vs 105.29 per 1000 PY, respectively), representing a covariate-adjusted HR of 0.30 (95% CI: 0.20–0.45). The magnitude of the association was statistically significant for male patients, who had an adjusted HR of 0.48 (95% CI: 0.39–0.58). Furthermore, the effect of CHM in reducing the risk of mortality was more prominent for females aged > 50 years (adjusted HR: 0.28; 95% CI: 0.17–0.44) (Table 3).

**Table 3:** Age- and sex-specific incidence and adjusted HR of death in relation to CHM among CHB patients with LCC

Variables	CHM nonusers			CHM users			Crude HR (95% CI)	Adjusted HR (95% CI)
	Case	PY	Incidence	Case	PY	Incidence		
Female								
≤ 50	24	475.39	50.48	8	647.20	12.36	0.26 (0.12–0.58)	0.41^*^ (0.17–0.75)
➢ 50	82	531.34	154.33	26	607.35	42.81	0.28 (0.18–0.45)	0.28^*^ (0.17–0.44)
All	106	1006.73	105.29	34	1254.55	27.10	0.26 (0.18–0.41)	0.30^ƪ^ (0.20–0.45)
Male								
≤ 50	165	1598.62	103.21	57	1378.04	41.36	0.40 (0.34–0.63)	0.48^*^ (0.35–0.61)
> 50	222	1392.63	159.41	65	1011.99	64.22	0.38 (0.35–0.59)	0.47^*^ (0.35–0.60)
All	387	2991.25	129.38	122	2390.03	51.04	0.39 (0.36–0.55)	0.48^ƪ^ (0.39–0.58)

Incidence rate is per 1000 PY.

^*^Model adjusted for urbanization level, monthly income, and CCI scores.

^ƪ^ Model adjusted for age, urbanization level, monthly income, and CCI scores.

The most commonly prescribed CHMs for patients with CHB and contaminant LC are summarized in Table 4. Among these products, ten formulae were found to lessen the subsequent risk of death, including Dan-shen, Yan-Hu-Suo, Bei-mu, Huang-Qin, Bie-Chia, Jia-Wei-Xiao-Yao-San, Xiao-Chai-Hu-Tang, Shu-Jing-Huo-Xue-Tang, Chai-Hu-Shu-Gan-Tang, and Long-Dan-Xie-Gan-Tang.

**Table 4:** Risk of death in relation to the top 10 used single herb and multi herb products for CHB patients with contaminant LC

Chinese herbal name	Scientific name	Frequency of prescriptions	Average duration (day)	Daily dose (g)	Crude HR (95% CI)	Adjusted HR^*^ (95% CI)
**Single-herb products**						
Dan-shen	Salvia miltiorrhiza Bunge., Rhizoma	19786	9.3	10.59	0.36(0.31-0.42)	0.34(0.29-0.40)
Yan-Hu-Suo	Corydalis yanhusuo W.T. Wang, Rhizoma	5915	9.2	10.62	0.28(0.20-0.39)	0.29(0.21-0.42)
Hai-Piao-Xiao	Endoconcha Sepiae, Cuttlefish Bone	3848	7.03	9.11	0.42(0.27-1.03)	0.46(0.30-1.05)
Bei-mu	Fritillaria thunbergii Miq., Balbus	3707	11.21	8.98	0.21(0.13-0.34)	0.22(0.13-0.35)
Huang-qi	Astragalus membranaceus (Fisch.) Bunge, Rhizoma	3616	13.42	17.73	0.40(0.12-1.09)	0.42(0.13-1.06)
Yu-Jin	Curcuma aromatica Salisb., Rhizoma	2959	7.69	7.42	0.38(0.04-1.02)	0.37(0.03-1.01)
Bai-Hua-She-She-Cao	Hedyotis diffusissima Merr., Herba	3384	12.00	16.10	0.49(0.19-1.22)	0.48(0.18-1.25)
Ge-gen	Pueraria lobata (Willd.) Ohwi, rhizoma	3412	8.35	9.76	0.42(0.11-1.20)	0.41(0.09-1.27)
Huang-Qin	Scutellaria baicalensis Georgi, Rhizoma	3374	7.47	8.80	0.19(0.12-0.30)	0.20(0.12-0.29)
Bie-Chia	Pelodiscus sinensis, soft-shelled turtle	2511	10.51	15.88	0.17(0.11-0.26)	0.18(0.12-0.27)
**Multi-herb products**						
Jia-wei-Xiao-Yao-San		6064	10.43	16.93	0.25(0.19-0.36)	0.26(0.18-0.36)
Xiao-Chai-Hu-Tang		4161	9.66	14.80	0.27(0.18-0.41)	0.27(0.19-0.40)
Shu-Jing-Huo-Xue-Tang		5297	7.47	11.92	0.22(0.15-0.33)	0.21(0.14-0.32)
Chai-Hu-Shu-Gan-Tang		3309	10.38	21.53	0.20(0.13-0.34)	0.22(0.13-0.35)
Ping-Wei-San		4803	8.22	16.85	0.50(0.30-1.45)	0.49(0.33-1.41)
Long-Dan-Xie-Gan-Tang		2864	8.25	15.01	0.31(0.21-0.48)	0.35(0.23-0.53)
Shao-Yao-Gan-Cao-Tang		3952	7.09	16.04	0.39(0.10-1.08)	0.41(0.13-1.12)
Xiang-Sha-Liu-Jun-Zi-Tang		3935	7.47	10.68	0.42(0.14-1.34)	0.46(0.15-1.36)
Yin-Chen-Wu-Ling-San		2603	6.88	11.26	0.36(0.10-1.01)	0.39(0.11-1.09)
Xue-Fu-Zhu-Yu-Tang		2697	7.44	11.55	0.34(0.08-1.11)	0.36(0.09-1.18)

^*^Model adjusted for age, gender, urbanization level, monthly income, and CCI scores.

## DISCUSSION

Hepatic encephalopathy, ascites, and esophageal variceal bleeding are 3 major cirrhosis-related complications. In clinical practice, the presence of any of these complications is associated with decompensated LC and is commonly predictive of a poor prognosis, such as death [3]. Therefore, identifying alternative treatments that can delay the progression of liver disease is important in the prevention of deaths among CHB patients with LC. In this study, we employed a nationwide population-based database to investigate the effect of CHM on the morality risk among these patients. To increase the diagnostic accuracy for patients who were diagnosed with CHB together with LC, all patients in our study were identified based on ICD-9-CM codes and if they applied for catastrophic illness certificate (CIC) card due to decompensated LC. This approach robustly increases the diagnostic accuracy of cirrhotic patients.

The effects of antiviral treatment for patients with CHB are well established [7, 8]. In this study, we further discovered that using CHM therapy during the standard antiviral treatment of CHB-related decompensated LC can lower the risk of mortality by 56%, which revealed the positive association of CHM and lowered risk of death among decompensated cirrhotic patients. Current medical guidelines have, in the past, been unsupportive of using CHM for CHB-related decompensated cirrhotic patients. However, over the past decade, certain CHM products have been commonly used for treating various illnesses [4–6]. A previous study conducted in Taiwan reported that the overall prevalence of insurance-covered traditional Chinese medicine (TCM) use in patients with liver cancer was as high as 21% [9]. In our previous study, we found that CHM users had a significantly lower HCC risk (37%) compared with non-CHM users [6]. LC involves a loss of normal liver structure that interrupts the normal blood flow in the liver and progresses to the nodules of the liver, thus resulting in functional failure [10]. We inferred that the possible pharmacologic mechanisms by which CHM treatment acts on CHB patients with LC includes antiviral effects [11], potent antioxidant activity, inhibition of free-radical–induced hepatic fibrosis by reducing cytochrome C [12], decreasing HBx-associated pathways [13], and modifying the microenvironment of HCC [14]. These effects of CHM treatments may contribute to the alleviation of liver fibrosis and cirrhosis.

In addition, results from our study indicate that elderly patients benefited the most from CHM in terms of reduced risk of death. No previous studies have been conducted to determine the long-term effect of CHM on the risk of death among CHB patients with contaminant LC, which precludes a comparison of results. Yet, several possible explanations may clarify these observed differences: First, the number of younger patients in our study was smaller, and the rates at which they developed adverse outcomes were somewhat lower than those of the older patients. Second, according to a previous study of the natural history of patients with CHB in Taiwan, the reported risk of LC in patients with CHB begins to rise during the patients’ 40s and increases significantly after age 50 [15]. Additionally, during 2000-2015, the death rates associated with LC in the US population increased 21% for men and 57% for women among persons aged 45–64 years [16]. Thus, the probability that LC risk increases with advanced age may explain the significant effect of CHM among elderly patients.

The present study has also listed the most commonly prescribed single herb and multi herb products in treating CHB patients with *contaminant LC*. Among the commonly prescribed multi-herb products, we noted that Jia-wei-Xiao-Yao-San (JWXYS) plays a role in lowering the risk of death. It could be argued that JWXYS which exhibits potent antioxidant activity and inhibits free-radical-induced hepatic fibrosis by cytochrome C reduction [12], also contributes to better prognosis for liver cancer patients. Chai-Hu-Shu-Gan-Tang (CHSGT) was also found to be significantly related to lower risk of death. We speculate that this herb could soothe the liver and disperse the stagnated liver Qi. It should be noted that the positive therapeutic effects derived from the components of CHSGT, such as Chai-Hu, Shao-Yao, Gan-Cao and Chuan-Xiong), were found to be identical to those of Si-Ni-San (SNS). Our previous study showed that SNS suppresses the HBx-induced invasiveness and metastatic potential of hepatocellular carcinoma cells [13]. This finding also suggests that CHSGT may reduce subsequent risk of death among the affected patients.

Another herbal formula that possesses liver protection, Xiao-Chai-Hu-Tang (XCHT), has been widely used in Asian to treat chronic viral liver diseases [6, 17, 18]. Chai-Hu (*Bupleurum chinense*) and Huang-qin (S*cutellaria baicalensis*) are two of the ingredients of XCHT. Saikosaponin-A is extracted from bupleurum, which acts as an antioxidant and suppresses inflammation and fibrogenesis [19]. Furthermore, several reports demonstrated that S*cutellaria baicalensis* and baicalein have anti-fibrotic effects on rat liver [20–22]. The beneficial effect of Long-Dan-Xie-Gan-Tang (LDXGT) in lowering risk of death was also found in the present study. This herbal product has been proven to decrease the serum lactate dehydrogenase (LDH) and ALT levels following liver injury and further reduce the cell degeneration and liver necrosis induced by CCl_4_, which in turn improves the biotransformation function of the liver [23]. A further finding indicated that Shu-Jing-Huo-Xue-Tang (SJHXT) was related to lower risk of death. The likely explanation for this finding is that SJHXT may have lessened the deterioration of hepatic injury by improving blood circulation and reducing the blood stasis syndrome [24].

Additionally, we also observed that those who used Yan-hu-suo were at a lower risk of death. This product is a well-known traditional CHM that was used for those with hepatitis or liver cirrhosis and acts to strengthen the immune system and to attenuate the pain sensory due to qi stagnation [25]. Another commonly used single drug prescription, Bei Mu, has been integrated into some Chinese herb formulae for treating cancer patients. The alkaloid verticinone, derived from Bei Mu, could exert an antinociceptive and anti-inflammatory effects on cancer-related neuropathic pain through both peripheral and central actions [26], which may enhance hepatoprotective activity.

Despite the clinical and research implications suggested by the present study, there are several limitations to consider. First, although the severity of LC was commonly based on the Child–Pugh score, it was difficult to identify the laboratory data or viral load in this database. Second, we identified patients with CHB and liver cirrhosis using ICD-9-CM codes, and misclassification may be possible. To decrease the possibility of this error, we selected patients with CHB only after they were recorded as having at least 3 outpatient visits that reported consistent diagnoses, or at least one inpatient admission. Otherwise, all patients with decompensated LC received a CIC from the National Health Insurance (NHI) program in Taiwan. For these patients, the diagnosis of decompensated LC was double-checked, which made the diagnosis of LC in this study more reliable. Third, during this study period, high-potency antiviral treatments for CHB such as entecavir or tenofovir were not widely used in Taiwan. However, this is the first nationwide population-based study that identifies the effect of CHM on the mortality rates of patients with CHB-related decompensated LC. Further study may be needed to more carefully evaluate the effects of CHM for patients with CHB-related cirrhosis, in particular if they also concurrently receive high-potency drugs. Fourth, evidence derived from any observational cohort study is generally less robust than data obtained from randomized trials because cohort study designs are subject to various biases, including confounding effects. Further research focusing on possible confounders identified in the literature are warranted to fully clarify the impact of CHM on the psychosocial health, especially on the patient's quality of life.

In conclusion, the use of CHM was associated with a significantly improved survival rate among CHB-related decompensated LC patients. The results of our study provide strong evidence for health care providers to consider the use of CHM therapy to improve the survive of patients with CHB-related decompensated LC.

## MATERIALS AND METHODS

### Data source

The NHI program of Taiwan was launched in 1995 and offers coverage to approximately 99% of the nation's population of 25 million [27]. For research and administrative purposes, the Taiwanese National Health Research Institute used these claims data to establish various database sets for public use. This retrospective cohort study used the Longitudinal Health Insurance Database (LHID) that comprises claims data for 1 million beneficiaries randomly sampled from the registry for beneficiaries of the National Health Insurance Research Database. Because a multistage stratified systematic sampling method was used, no statistically significant differences were observed in age, sex, or average salary between the sample group and all enrollees in the NHI program [27]. This dataset contains information about all medical services, such as ambulatory care claims, inpatient claims, prescription drugs, and claimed medical expenses. To date, > 300 published scientific papers have been based on this database [28].

This study was conducted in accordance with the Helsinki Declaration, and it was also evaluated and approved by the local Institutional Review Board and Ethics Committee of Buddhist Dalin Tzu Chi Hospital, Taiwan (No. B10004021-1). Because the LHID files contain only de-identified secondary data, the review board waived the requirement for obtaining informed consent from the patients.

### Study population

**Figure 2:**
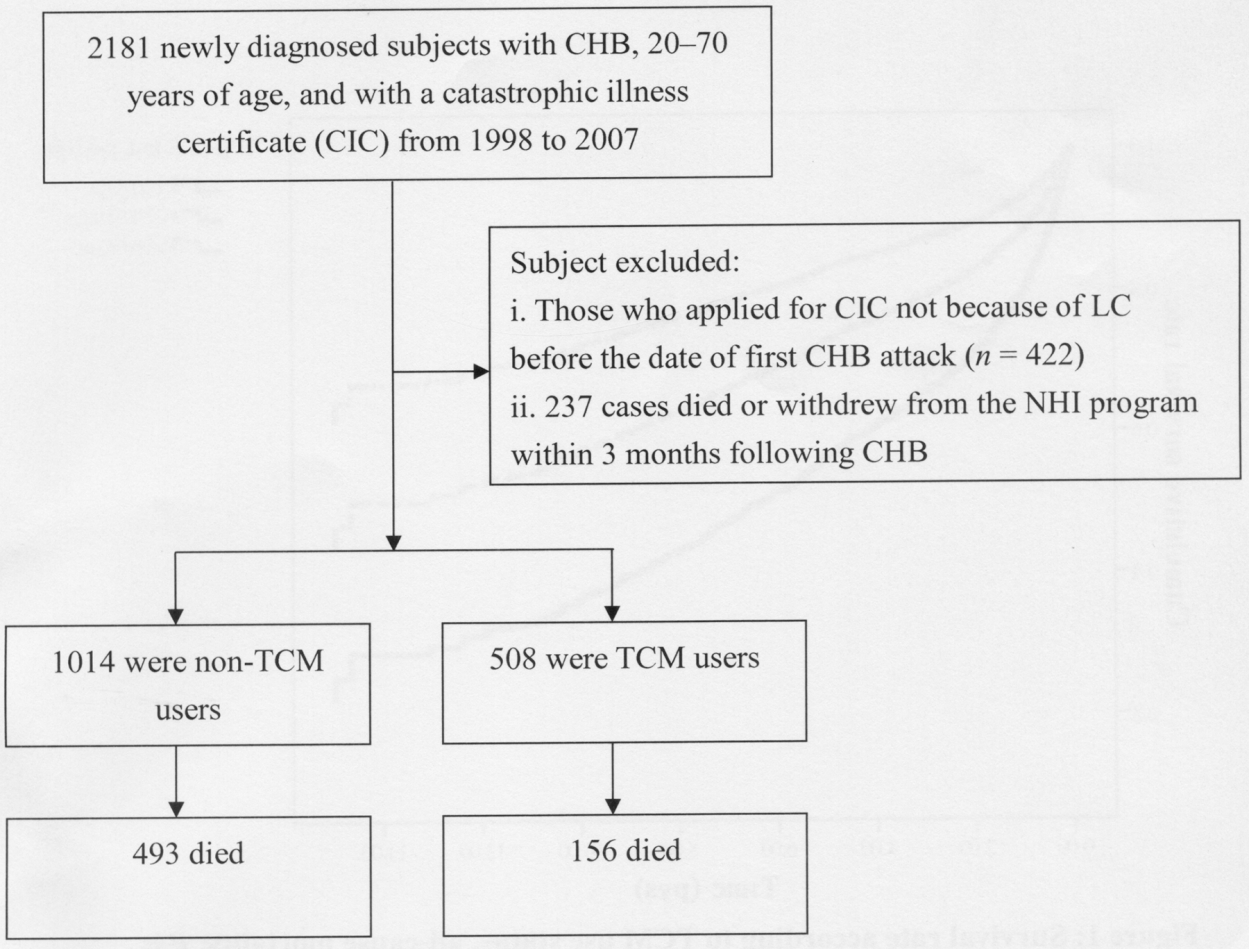
Flowchart of selection and follow-up of study subjects

The participant selection process is shown in Figure 2. All diagnoses in this insurance claims dataset were coded using the *International Classification of Disease, 9th Revision, Clinical Modification* (*ICD-9-CM*). With it, we identified patients 20–to-70 years of age with newly diagnosed CHB during 1998–2007 (ICD-9-CM codes: 070.2, 070.3, or V02.61) and treatment with nucleos(t)ide analogues (lamivudine, adefovir, telbivudine, entecavir, or tenofovir) as the study cohort. For an accurate diagnosis of CHB, the patients who received at least 3 diagnoses during outpatient visits or who were admitted to a hospital with a primary diagnosis of CHB within the observational period were selected. The index event was defined as the first attack of CHB. Thereafter, among all subjects we enrolled only those who applied for a CIC due to the accompanying LC. In Taiwan, insured residents with major diseases (eg, cancer, chronic mental illness, end-stage renal disease, several autoimmune diseases) can apply for the CIC card and are not required to make copayments when seeking healthcare for catastrophic illness. Initially, 2181 CHB patients with the CIC card were recruited. However, 422 cases were excluded because they applied for CIC for reasons other than LC. Additionally, patients who died or were followed for < 3 months after the first CHB diagnosis were excluded (*n* = 237). The outcome of interest, all-cause mortality, was derived from CIC because an item within this record notes the death date if the patient has died. Finally, a total of 1522 subjects were included in the data analysis.

In Taiwan, only certified Chinese medicine physicians are entitled to provide CHM therapy. We used the frequency of visits for TCM to verify the CHM exposure of each study subject. Patients who received CHM treatment for > 30 days were considered CHM users, whereas those who were treated for ≤ 30 days were considered non-CHM users [[4 6]]. The index date of the follow-up period for those who were classified as non-CHM users was assigned to begin on the date of the first CHB diagnosis, and the index date for the follow-up period for CHB cases with CHM use was assigned as the date of the initiation of CHM services. All patients were followed from the index date to the date of death, withdrawal from the insurance program, or the end of 2012, whichever came first.

### Demographic characteristics and comorbidities

Demographic characteristics evaluated in this study included age, sex, income for estimating insurance payments, and urbanization level of patients’ residential areas. Patients’ monthly incomes were stratified into 3 levels: ≤ New Taiwan Dollar (NTD) 17,880, NTD 17,881–NTD 43,900, and ≥ NTD 43,901. Urbanization levels were divided into 3 strata by population: urban (levels 1–2), suburban (levels 3–4), and rural (levels 5–7) areas. Level 1 refers to the “most urbanized,” and level 7 refers to the “least urbanized” communities [29]. We also employed the Charlson–Deyo comorbidity index [30] to control for the overall disease burden of various comorbidities based on each study participant's medical records 1 year prior to cohort entry.

### Statistical analysis

The characteristics of the cohort at baseline were compared by *t*-tests and χ^2^ tests. The incidence rate of mortality between 2 groups is the number of cases per 1000 person–years. Hazard ratios and 95% confidence intervals (CI) were derived from Cox proportional hazards regression and met the assumption of proportionality of risks. To adjust for potential confounding in the relationship between the baseline characteristics and the risk of mortality, we further applied multivariate analysis, using Cox regression to rule out the actual effect of CHM on the risk of all-cause mortality. In addition, to test the robustness of the relation between CHM use and the subsequent risk of death, we divided CHM users into 2 subgroups: 1 group used CHM for 30–180 days, and the other group used CHM for > 180 days. Kaplan–Meier survival curves were generated for overall survival probability according to 2 subtypes with respect to CHM use. The Wilcoxon log-rank test was used to examine for differences. Furthermore, if an interaction of age and sex occurred with respect to the risk of death, a stratified analysis by 2 factors was conducted to more accurately determine the relative risk of mortality between CHM users and non-CHM users. All statistical analyses were conducted using SAS version 9.3 (SAS Institute Inc., Cary, NC, USA). A *P* value of < 0.05 was considered to be statistically significant.

## Abbreviations

CHB: Chronic hepatitis B
LC: Liver cirrhosis
CHM: Chinese herbal medicine
HBV: Hepatitis B virus
HCC: Hepatocellular carcinoma
PY: Person-years
HR: Hazard ratio
CI: Confidence interval
CIC: Catastrophic illness certificate
NHI: National health insurance
LHID: Longitudinal health insurance database
TCM: Traditional Chinese medicine
ICD-9-CM: International Classification of Disease, 9th revision
NTD: New Taiwan dollar
